# Supplementary material for: Analysis of PPARγ Signaling Activity in Psoriasis
Source: Int J Mol Sci. 2021 Aug 10;22(16):8603. doi: 10.3390/ijms22168603 (PMC8395241; doi:10.3390/ijms22168603)
Supplement: Supplementary file 1 [file ijms-22-08603-s001.zip › Supplemental materials_Analysis of PPARg signaling activity in psoriasis/Pathway models/Models images and html files/Model of PPARG related pathways in psoriasis (short version)/38049.html]

IRAK4


# Protein IRAK4

|  |  |
| --- | --- |
| URN | urn:agi-llid:51135 |
| Total Entities | 0 |
| Connectivity | 2141 |
| Name | IRAK4 |
| Description | interleukin-1 receptor-associated kinase 4 |
| Notes | This gene encodes a kinase that activates NF-kappaB in both the Toll-like receptor (TLR) and T-cell receptor (TCR) signaling pathways. The protein is essential for most innate immune responses. Mutations in this gene result in IRAK4 deficiency and recurrent invasive pneumococcal disease. Multiple transcript variants encoding different isoforms have been found for this gene. [provided by RefSeq, Aug 2011] |
| Primary Cell Localization | Cytoplasm |
| Class | Protein kinase |
| ObjectType | Protein |

---

|  |  |
| --- | --- |
| Pathway | Osteoarthritis |
|  | Fatty Acids Block TLR Signaling in Enteroendocrine Cell |
|  | I-cell: CCK-8 Secretion and Regulation of Eating Behavior |
|  | Toll-like Receptors Act through MYD88-TIRAP Signaling |
|  | Antiviral Signaling through Pattern Recognition Receptors |
|  | Toll-like Receptors Act through MYD88 Signaling |
|  | TLR4 Signaling in Leukocytes |
|  | Th1-Cell Differentiation |
|  | Th17-Cell Differentiation |
|  | Toll-like Receptors in Sterile Inflammation |
|  | Toll-like Receptor Independent Sterile Inflammation |
|  | Basophil Activation |
|  | Eosinophil Survival by Cytokine Signaling |
|  | Mast-Cell Activation without Degranulation |
|  | Mast-Cell Activation without Degranulation through IL33/IL1RL1 Signaling |
|  | Mast-Cell Activation without Degranulation through IL1R1 and TLR Signaling |
|  | Macrophage M1 Lineage |
|  | CD16/CD14 Proinflammatory Monocyte Activation |
|  | AHR Signaling in M1 Macrophages Function |
|  | anti-Inflammatory Function of Macrophage M2 Lineage |
|  | AXL Receptor Inhibits Macrophages and Dendritic Cells Function |
|  | Neutrophil Recruitment and Priming |
|  | Negative Acute Phase Proteins Synthesis |
|  | Positive Acute Phase Proteins Synthesis |
|  | Vascular Endothelial Cell Activation by Cytokines |
|  | Vascular Endothelial Cell Activation by Blood Coagulation Factors |
|  | Adipokines Production by Adipocyte |
|  | IL1B Expression Targets -> Nociception |
|  | Proteins Involved in Atherosclerosis |
|  | Dendritic Cells Function in Atherosclerosis |
|  | RAGE/AGER and S100 Proteins in Cardiovascular Injury |
|  | Th1-Cells Activation and Proliferation in Atherosclerosis |
|  | Myocardial Infarction |
|  | Proteins Involved in Myocardial Ischemia |
|  | Lymphocyte Mediated Myocardial Injury in Myocarditis |
|  | Proteins Involved in Myocarditis |
|  | Toll-like Receptors (TLR) in Antiviral Innate Immune Response |
|  | Inflammatory Reaction in Acne Vulgaris |
|  | Keratinocyte Proliferation in Acne Vulgaris |
|  | Mast-Cells Activation in Atopic Dermatitis |
|  | Dendritic Cells Function in Psoriasis |
|  | Apoptotic Keratinocytes Clearance Recession in Systemic Lupus Erythematosus |
|  | Dendritic Cell Activation in Systemic Lupus Erythematosis |
|  | Th17-Cell Function in Systemic Lupus Erythematosus |
|  | Neutrophil and Macrophage Function in Systemic Lupus Erythematosus |
|  | Vitiligo |
|  | T-Cell Cytotoxic Response against Melanocytes in Vitiligo |
|  | Plasmacytoid Dendritic Cell in Diabetes Mellitus Type 1 |
|  | TLR2 Signaling in Treg-Cell in Type 1 Diabetes (Animal Model) |
|  | Toll-like Receptors in beta-Cell |
|  | TLR7 and TLR9 in Plasmacytoid Dendritic Cell |
|  | Conventional Dendritic Cell Role in Diabetes Mellitus Type 1 |
|  | Insulin Resistance in Myocytes Induced by Obesity |
|  | Hepatic Stellate Cells in non-Alcoholic Fatty Liver Disease |
|  | beta-Cell Destruction through Cytokines in Diabetes Mellitus |
|  | beta-Cell Destruction in Diabetes Mellitus Type 1 |
|  | Adipokines Production by Adipocyte Impaired in Obesity |
|  | CD4+T-Cell Response in Celiac Disease |
|  | Celiac Disease |
|  | Crohn's Disease |
|  | Dendritic Cell Dysfunction in Crohn's disease |
|  | Dendritic Cell Function in Ulcerative Colitis |
|  | Epithelial Cell in the Innate Immune Response in Ulcerative Colitis |
|  | Nicotine anti-Inflammatory Effect in Ulcerative Colitis |
|  | Antiphospholipid Antibodies in Endothelial Cell |
|  | B-Cell Chronic Lymphocytic Leukemia |
|  | Diffuse Large-B-Cell Lymphoma |
|  | Diffuse Large-B-Cell Lymphoma ABC Subtype |
|  | Mucosa-Associated Lymphoid Tissue (MALT) Lymphoma |
|  | Splenic Marginal Zone Lymphoma |
|  | Antiphospholipid Antibodies and Endothelial Cell Interaction |
|  | IL33 Signaling Related Eosinophilia in Asthma |
|  | TSLP Signaling in Bronchial Epithelial Cell in Asthma |
|  | Club-Cell Role in Asthma |
|  | Basophil Activation in Asthma |
|  | Antigen-Presenting Cell Role in Asthma |
|  | Th17-Cell Differentiation in Asthma |
|  | Mucin Production in Goblet Airway Epithelial Cells |
|  | CFTR Facilitates Oxidative Stress in Airway Epithelium in CF |
|  | Airway Epithelial Cell Dysfunction |
|  | CFTR Expression in Epithelial Cells (Class I Mutations) |
|  | Osteoclast Activation in Gout |
|  | Neutrophil Recruitment in Sinovium in Gout |
|  | Monocytes Function in Gout |
|  | TNF and IL1B Induce Metalloproteinase Synthesis in Osteoarthritis |
|  | IL1B Induced Arthralgia |
|  | Dendritic Cell Dysfunction in Psoriatic Arthritis |
|  | Th17-Cell and Th1 Immune Responsein Psoriatic Arthritis |
|  | Osteoclast Activation in Psoriatic Arthritis |
|  | Synovial Fibroblast Activation in Psoriatic Arthritis |
|  | Osteoclast Activation in Rheumatoid Arthritis |
|  | Synovial Fibroblast Activation by Citokines in Rheumatoid Arthritis |
|  | TLR2 Induced Synovial Fibroblast Activation in Rheumatoid Arthritis |
|  | Autocrine Cytokine/Chemokine Loops in Systemic Scleroderma |
|  | Th17-Cell Numbers Reduction in HIV |
|  | Toll-like Receptors (TLR) in Periodontitis |
|  | IL1 Receptor Antagonist (IL1RN) Deficiency (DIRA) |
|  | PAPA Syndrome |
|  | TNF Receptor Associated Periodic Syndrome |
|  | Mevalonate Kinase Deficiency |
|  | Blau Syndrome |
|  | IL36 Receptor Antagonist (IL1F5) Deficiency (DITRA) |
|  | Cryopyrin (NLPR3) Associated Periodic Syndromes |
|  | Familial Cold Autoinflammatory Syndrome 2 |
|  | Familial Mediterranean Fever |
|  | Majeed Syndrome |
|  | Gluten Impact on Neuronal System (Hypothesis) |
|  | Microglia Activation in Alzheimer's Disease |
|  | HMGB1 and IL1B in Neuroinflammation |
|  | Microglia Activation in Parkinson's Disease |
|  | Subepithelial Fibroblasts in Middle Ear Cholesteatoma |
|  | Cholesteatoma |
|  | HPV E6 and E7 Induced Disruption of TNFR/NF-kB Signaling in Keratinocytes |
|  | Cytokines Trigger Otitis Media |
|  | MUC5AC Expression by NTHi and S. Pneumoniae |
|  | Immune System Role in Otitis Media |
|  | Alveolar Macrophage Negative Regulation Declined in COPD |
|  | Alveolar Macrophages Dysfunction in COPD |
|  | T-Cell Cytotoxic Activation |
|  | Alveolar Epithelial Cell Dysfunction in COPD |
|  | Mucin Hyperproduction in Goblet and Mucous Cells |
|  | Endothelial Cell Dysfunction in Glomerulonephritis |
|  | Toll-like Receptors in Pyelonephritis |
|  | Endothelial Cell Dysfunction in Pyelonephritis |
|  | NFKB Canonical Signaling Activation in Cancer |
|  | STAT3 and NFkB Activate Inflammation-Induced Tumorigenesis |
|  | NFKB Signaling Activation by Blocking of Tumor Suppressors |
|  | IDO1 in Cancer Immune Escape |
|  | IL1R -> STAT3 Signaling |
|  | IL1R -> NF-kB Signaling |
|  | NF-kB Canonical Signaling |
|  | TLR4 -> AP-1 Expression Targets |
|  | TLR5 Expression Targets |
|  | TLR7 Expression Targets |
|  | TLR4 -> AP-1/EGR1/HIF1A Expression Targets |
|  | TLR1 -> 2/6 Expression Targets |
|  | IL1B Expression Targets |
|  | TLR4 -> NF-kB/IRF Expression Targets |
|  | IL1A Expression Targets |
|  | TLR9 Expression Targets |
|  | TLR4 -> IRF Signaling |
|  | TLR4/5/7/9 -> NF-kB Signaling |
|  | TLR1/2/6 -> NF-kB Signaling |
|  | TLR -> AP-1 Signaling |
|  | pathway 4 |
|  | Model of PPARG signaling in psoriasis |
|  | Model of PPARG related pathways in psoriasis (short version) |
|  | dsRNA induces innate immunity and INFs in alveolar cells |
|  | Innate immunity in Leukocytes |
|  | AGER feed forward loop in innate immunity |
|  | Dendritic Cell Activation in COVID19 |
|  | 1\_Inflammation in keratinocytes in the pathology of acne |
|  | 2\_Acute phase initiation in atopic dermatitis |
|  | 1\_TNF and IL1B provoke ECM degradation in osteoarthritis |
|  | 2\_Chondrocyte death in osteoarthritis |
|  | 1\_The role of Toll-like receptors in pyelonephritis |
|  | 2\_The role of endothelial cells in pyelonephritis |
|  | 1\_Insufficient activation of immune response in the middle ear epithelium cells in otitis media |
|  | 2\_Pathogens stimulate mucins expression in the middle ear |
|  | 3\_Arterial wall inflammation and calcification in atherosclerosis |
|  | 1\_Cigarette smoke causes dysfunction and production of inflammatory mediators in alveolar epithelial cells |
|  | 3\_Role of alveolar macrophages in pulmonary tissue destruction in COPD |
|  | 1\_Th2 cell response in asthma |
|  | 3\_1\_Mucus accumulation in asthma |
|  | 2\_2\_Eosinophilia and neutrophilia in asthma: eosinophil apoptosis |
|  | 4\_Mucin hyperproduction in goblet and mucous cells in COPD |
|  | 1\_Defects in response to pathogens in the gut promotes inflammation in Crohn's disease |
|  | 2\_Intestine epithelial cells drive immunological responses in ulcerative colitis |
|  | 1\_Intestine epithelial cell dysfunction in ulcerative colitis |
|  | 2\_Lipotoxicity in NAFLD |
|  | 1\_CD4+ T cell and epithelial cell response in celiac disease |
|  | 5\_Human papilloma virus E6 and E7 proteins promote epithelial cell proliferation |
|  | 4\_3\_Insulin resistance: FFA-related insulin resistance |
|  | 1\_CFTR expression and degradation in epithelial cells in cystic fibrosis (class I-II mutations) |
|  | 1\_Antigen-presenting cells promote islet dysfunction and immune system activation in T1D |
|  | 3\_Islet beta-cell destruction in T1D |
|  | 2\_Neuron and Schwann cell death |
|  | Figure 4\_Diabetes mellitus Type 1: conventional dendritic cell |

---

|  |  |
| --- | --- |
| MedScan ID | 51135 |

---

|  |  |
| --- | --- |
| LocusLink ID | 51135 |
|  | 266632 |
|  | 300177 |
|  | 71485 |
|  | 77562 |
|  | 108842 |
|  | 239628 |

---

|  |  |
| --- | --- |
| Alias | RIKEN cDNA 9330209D03 gene |
|  | RIKEN cDNA 8430405M07 gene |
|  | OTTMUSP00000018066 |
|  | LOC239628 |
|  | interleukin-1 receptor associated kinase 4 |
|  | IL-1 receptor associated kinase 4 |
|  | 9330209D03Rik |
|  | renal carcinoma antigen NY-REN-64 |
|  | IRAK4 |
|  | IL-1R associated kinase (IRAK) 4 |
|  | NY REN 64 antigen |
|  | REN64 |
|  | 9330209D03 |
|  | 8430405M07 |
|  | IPD1 |
|  | IL-1R associated kinase 4 |
|  | IL-1 receptor-associated kinase (IRAK) IV |
|  | 8430405M07Rik |
|  | interleukin-1 receptor associated kinase (IRAK) 4 |
|  | interleukin-1 receptor-associated kinase (IRAK) IV |
|  | LOC65651 |
|  | IRAK-4 mutated form 1 |
|  | IL-1 receptor associated kinase (IRAK) 4 |
|  | OTTMUSP00000018065 |
|  | IL-1 receptor-associated kinase IV |
|  | IL-1R-associated kinase IV |
|  | LOC95458 |
|  | NY-REN-64 |
|  | IL-1R-associated kinase (IRAK) IV |
|  | interleukin-1 receptor-associated kinase IV |
|  | LOC51135 |
|  | IRAK-4 |
|  | interleukin-1 receptor-associated kinase 4 |
|  | IRAK |

---

|  |  |
| --- | --- |
| EC Number | 2.7.11.1 |

---

|  |  |
| --- | --- |
| GO ID | 0005524 |
|  | 0005149 |
|  | 0000287 |
|  | 0004672 |
|  | 0004674 |
|  | 0007254 |
|  | 0002755 |
|  | 0001816 |
|  | 0019221 |
|  | 0045087 |
|  | 0070498 |
|  | 0035556 |
|  | 0002446 |
|  | 1990266 |
|  | 0043123 |
|  | 0051092 |
|  | 0048661 |
|  | 0034162 |
|  | 0002224 |
|  | 0005737 |
|  | 0005829 |
|  | 0010008 |
|  | 0005615 |
|  | 0005634 |
|  | 0005886 |
|  | 0016301 |
|  | 0000166 |
|  | 0016740 |
|  | 0002376 |
|  | 0016310 |
|  | 0006468 |
|  | 0007165 |
|  | 0005622 |
|  | 0034146 |
|  | 0034166 |
|  | 0038123 |
|  | 0045944 |
|  | 0016772 |
|  | 0038124 |
|  | 0034142 |
|  | 0034134 |
|  | 0008063 |
|  | 0034130 |
|  | 0004872 |
|  | 0007249 |
|  | 0005575 |
|  | 0005515 |
|  | 0003674 |
|  | 0004713 |

---

|  |  |
| --- | --- |
| KEGG ID | hsa:51135 |
|  | mmu:266632 |
|  | rno:300177 |

---

|  |  |
| --- | --- |
| MedLine Reference | 10508479 |
|  | 11923871 |
|  | 11960013 |
|  | 12297423 |
|  | 12477932 |
|  | 12496252 |
|  | 12637671 |
|  | 12721283 |
|  | 12860405 |
|  | 12925671 |
|  | 14702039 |
|  | 15084582 |
|  | 15292196 |
|  | 15489334 |
|  | 15825022 |
|  | 15905496 |
|  | 16286015 |
|  | 16286016 |
|  | 17046325 |
|  | 17141195 |
|  | 17161373 |
|  | 17217339 |
|  | 3772297 |
|  | 10349636 |
|  | 11042159 |
|  | 11076861 |
|  | 11217851 |
|  | 12466851 |
|  | 16141072 |
|  | 16141073 |
|  | 11544199 |
|  | 12538665 |
|  | 12682231 |
|  | 12819136 |
|  | 14634120 |
|  | 16040976 |
|  | 16177054 |
|  | 16179420 |
|  | 16574867 |
|  | 17178921 |
|  | 17337443 |
|  | 17360955 |
|  | 15890643 |

---

|  |  |
| --- | --- |
| Organism | Homo sapiens {Organism urn:agi-taxid:9606} |
|  | Mus musculus {Organism urn:agi-taxid:10090} |
|  | Rattus norvegicus {Organism urn:agi-taxid:10116} |
|  | Homo sapiens |
|  | Mus musculus |
|  | Rattus norvegicus |

---

|  |  |
| --- | --- |
| Mouse chromosome position | 15 |
|  | 15 F1 |

---

|  |  |
| --- | --- |
| OMIM ID | 606883 |
|  | 610799 |
|  | 607676 |

---

|  |  |
| --- | --- |
| Rat chromosome position | 7q35 |

---

|  |  |
| --- | --- |
| Hugo ID | 17967 |
|  | HGNC:17967 |

---

|  |  |
| --- | --- |
| Human chromosome position | 12q12 |

---

|  |  |
| --- | --- |
| Swiss-Prot Accession | B4E359 |
|  | Q69FE3 |
|  | Q9NWZ3 |
|  | B2RAP9 |
|  | Q9NWZ3.1 |
|  | Q8R4K2 |
|  | Q8R4K2.1 |
|  | D4A7K4 |
|  | Q69FE1 |
|  | Q8TDF7 |
|  | Q9Y589 |
|  | Q80WW1 |
|  | Q7Z6A7 |
|  | Q7Z6A8 |
|  | Q3USX5 |
|  | Q9D250 |

---

|  |  |
| --- | --- |
| GenBank ID | NC\_000012 |
|  | XM\_017019390 |
|  | XP\_016874879 |
|  | NM\_001351338 |
|  | NP\_001338267 |
|  | NM\_001351345 |
|  | NP\_001338274 |
|  | NM\_001351341 |
|  | NP\_001338270 |
|  | NM\_001351343 |
|  | NP\_001338272 |
|  | NM\_001351339 |
|  | NP\_001338268 |
|  | NM\_001114182 |
|  | NP\_001107654 |
|  | NM\_001145256 |
|  | NP\_001138728 |
|  | NM\_001351340 |
|  | NP\_001338269 |
|  | NM\_016123 |
|  | NP\_057207 |
|  | NM\_001145257 |
|  | NP\_001138729 |
|  | NM\_001351344 |
|  | NP\_001338273 |
|  | NM\_001351342 |
|  | NP\_001338271 |
|  | NM\_001145258 |
|  | NP\_001138730 |
|  | XM\_024449008 |
|  | XP\_024304776 |
|  | XM\_006719440 |
|  | XP\_006719503 |
|  | XM\_006719442 |
|  | XP\_006719505 |
|  | XM\_005268943 |
|  | XP\_005269000 |
|  | XM\_005268949 |
|  | XP\_005269006 |
|  | XM\_006719438 |
|  | XP\_006719501 |
|  | XM\_005268945 |
|  | XP\_005269002 |
|  | XM\_011538431 |
|  | XP\_011536733 |
|  | XM\_005268944 |
|  | XP\_005269001 |
|  | XM\_011538433 |
|  | XP\_011536735 |
|  | NG\_009892 |
|  | AC093012 |
|  | AX431306 |
|  | CAD37279 |
|  | AY186092 |
|  | AAN75440 |
|  | CH471111 |
|  | EAW57867 |
|  | AF155118 |
|  | AAD42884 |
|  | AF445802 |
|  | AAM15772 |
|  | AI126481 |
|  | AK000528 |
|  | BAA91232 |
|  | AK027301 |
|  | AK299944 |
|  | BAG61774 |
|  | AK304583 |
|  | BAG65371 |
|  | AK314288 |
|  | BAG36946 |
|  | AY283670 |
|  | AAP57089 |
|  | AY283671 |
|  | AAP57090 |
|  | AY340962 |
|  | AAR02358 |
|  | AY340963 |
|  | AAR02359 |
|  | AY340964 |
|  | AAR02360 |
|  | AY340965 |
|  | AAR02361 |
|  | AY340966 |
|  | AAR02362 |
|  | AY340967 |
|  | AAR02363 |
|  | AY730682 |
|  | AAX22228 |
|  | BC013316 |
|  | AAH13316 |
|  | DA290809 |
|  | KU178631 |
|  | ALQ34089 |
|  | KU178632 |
|  | ALQ34090 |
|  | Q9NWZ3 |
|  | NC\_000081 |
|  | XM\_011245645 |
|  | XP\_011243947 |
|  | NM\_029926 |
|  | NP\_084202 |
|  | AC147160 |
|  | CH466550 |
|  | EDL04264 |
|  | EDL04265 |
|  | AF445803 |
|  | AAM15773 |
|  | AK020397 |
|  | BAB32090 |
|  | AK028837 |
|  | BAC26146 |
|  | AK029028 |
|  | AK078803 |
|  | AK139992 |
|  | BAE24205 |
|  | BC051676 |
|  | AAH51676 |
|  | BE635313 |
|  | Q8R4K2 |
|  | NC\_005106 |
|  | NM\_001106791 |
|  | NP\_001100261 |
|  | XM\_006242193 |
|  | XP\_006242255 |
|  | AC\_000075 |
|  | AABR07058739 |
|  | AABR07058740 |
|  | AAHX01052385 |
|  | CH474027 |
|  | EDL76640 |
|  | EDL76641 |
|  | EDL76642 |
|  | XM\_011538432 |
|  | XM\_005268946 |
|  | XM\_005268947 |
|  | XM\_005268948 |
|  | AAHY01124586 |
|  | AAHY01124585 |
|  | AAHY01124584 |
|  | XP\_006719506 |
|  | XP\_006719504 |
|  | XP\_006719502 |
|  | ABM87133 |
|  | AC\_000037 |
|  | AABR06052501 |
|  | AABR06052500 |
|  | DQ892885 |
|  | XP\_005269005 |
|  | XP\_005269004 |
|  | XP\_011536734 |
|  | XP\_005269003 |
|  | AC\_000144 |
|  | NC\_018923 |
|  | LK938723 |
|  | AMYH02026382 |
|  | ABM83811 |
|  | AABR06052502 |
|  | XM\_006719443 |
|  | DQ896134 |
|  | XM\_006719439 |
|  | ABBA01031423 |
|  | XM\_006719441 |
|  | ABBA01031422 |
|  | NT\_029419 |
|  | NW\_925351 |
|  | NW\_001838057 |
|  | AC\_000055 |
|  | Q69FE1 |
|  | Q69FE3 |
|  | Q7Z6A7 |
|  | Q7Z6A8 |
|  | NT\_039621 |
|  | NW\_001030577 |
|  | Q3USX5 |
|  | Q9D250 |
|  | NW\_047783 |
|  | NW\_001084860 |
|  | XM\_001058330 |
|  | XP\_217026 |
|  | XM\_217026 |
|  | XP\_001058330 |
|  | 119578271 |
|  | 123987595 |
|  | 123999100 |
|  | 15426432 |
|  | 20219010 |
|  | 26000793 |
|  | 31871818 |
|  | 31871820 |
|  | 37727959 |
|  | 37727961 |
|  | 37727963 |
|  | 37727965 |
|  | 37727967 |
|  | 37727969 |
|  | 45220438 |
|  | 50401181 |
|  | 5360131 |
|  | 60502299 |
|  | 7020684 |
|  | 74708987 |
|  | 74713733 |
|  | 74738925 |
|  | 74758864 |
|  | 74759223 |
|  | BAD12234 |
|  | Q6ZWT8 |
|  | 15919912 |
|  | 123785112 |
|  | 148672317 |
|  | 148672318 |
|  | 20219012 |
|  | 26324784 |
|  | 30354528 |
|  | 50401077 |
|  | 74145960 |
|  | 81881248 |
|  | BM220651 |
|  | 149017636 |
|  | 149017637 |
|  | 149017638 |

---

|  |  |
| --- | --- |
| Swiss-Prot ID | IRAK4\_HUMAN |
|  | IRAK4\_MOUSE |
|  | B2RAP9\_HUMAN |
|  | Q69FE3\_HUMAN |
|  | B4E359\_HUMAN |
|  | D4A7K4\_RAT |
|  | Q69FE1\_HUMAN |

---

|  |  |
| --- | --- |
| Cell Localization | Cytoplasm |

---

|  |  |
| --- | --- |
| Ensembl ID | ENSG00000198001 |
|  | ENSP00000446490.1 |
|  | ENST00000551736.5 |
|  | ENSP00000390327.1 |
|  | ENST00000431837.5 |
|  | ENSP00000408734.2 |
|  | ENST00000440781.6 |
|  | ENSP00000479889.2 |
|  | ENST00000613694.4 |
|  | ENSP00000390651.3 |
|  | ENST00000448290.6 |
|  | ENSMUSG00000059883 |
|  | ENSMUSP00000074471.3 |
|  | ENSMUST00000074936.9 |
|  | ENSRNOG00000005965 |
|  | ENSRNOP00000007932.4 |
|  | ENSRNOT00000007932.5 |
|  | ENST00000551736 |
|  | ENSMUST00000074936 |
|  | ENSMUSP00000074471 |
|  | ENSP00000408734 |
|  | ENSP00000390327 |
|  | ENST00000431837 |
|  | ENST00000613694 |
|  | ENSRNOP00000007932 |
|  | ENSRNOT00000007932 |
|  | ENST00000440781 |
|  | ENSP00000446490 |
|  | ENSP00000479889 |

---

|  |  |
| --- | --- |
| MGI ID | MGI:2182474 |
|  | 2182474 |
|  | 1918735 |
|  | 1924812 |

---

|  |  |
| --- | --- |
| RGD ID | 1305303 |

---

|  |  |
| --- | --- |
| Unigene ID | Rn.61427 |
|  | Hs.138499 |
|  | Mm.422858 |

---

|  |  |
| --- | --- |
| Homologene ID | 41109 |

---

|  |  |
| --- | --- |
| Shape | Sickle-vertex |

---

|  |  |
| --- | --- |
| IPI ID | IPI00153899 |
|  | IPI00007641 |
|  | IPI00798404 |
|  | IPI00372984 |
|  | IPI00385374 |
|  | IPI00460849 |
|  | IPI00755511 |
|  | IPI00188049 |

---

|  |  |
| --- | --- |
| Homo sapiens Chromosome position | 12q12 |

---

|  |  |
| --- | --- |
| FunctionalClass | ATP binding |
|  | kinase |
|  | magnesium ion binding |
|  | nucleotide binding |
|  | protein binding |
|  | protein serine/threonine kinase |
|  | protein-tyrosine kinase |
|  | receptor |
|  | transferase |
|  | interleukin-1 receptor binding |
|  | protein kinase |
|  | molecular\_function |

---

|  |  |
| --- | --- |
| Mus musculus Chromosome position | 15 E3 |
|  | 15 F1 |

---

|  |  |
| --- | --- |
| KEGG pathway | Apoptosis |
|  | Toll-like receptor signaling pathway |

---

|  |  |
| --- | --- |
| Rattus norvegicus Chromosome position | 7q35 |

---

|  |  |
| --- | --- |
| Ensembl Gene ID | LRG\_75 |
|  | ENSG00000198001 |

---
